# Do people with musculoskeletal pain differ from healthy cohorts in terms of global measures of strength? A systematic review and meta-analysis

**DOI:** 10.1177/02692155221128724

**Published:** 2022-09-25

**Authors:** Enrico Verdini, Luca Maestroni, Michael Clark, Anthony Turner, Jörg Huber

**Affiliations:** 1 School of Health Sciences, 171115 University of Brighton, Brighton, UK; 2 Studio Medico Jacini, Rome, Italy; 3 London Sport Institute, 244879 School of Science and Technology, 389681 Middlesex University, London, UK; 4 Independent Consultant, Cambridge, UK

**Keywords:** Muscle strength assessment, multi-joint exercises, musculoskeletal pain, systematic review, meta-analysis

## Abstract

**Objective:**

It is currently unknown if people with musculoskeletal pain display different multi-joint strength capacities than healthy cohorts. The aim was to investigate whether people with musculoskeletal pain show differences in global measures of strength in comparison to healthy cohorts.

**Data sources:**

A systematic review was conducted using three databases (Medline, CINAHL and SPORTDiscus) and Preferred Reporting Items for Systematic Reviews and Meta-Analyses guidelines.

**Review methods:**

Studies involving participants with painful musculoskeletal conditions and multi-joint strength assessment measured at baseline were included. A meta-analysis was also performed to compute standardized mean differences (± 95% confidence intervals), using Hedge's *g*, and examined the differences in multi-joint strength at baseline between participants with painful musculoskeletal conditions and healthy participants.

**Results:**

In total, 5043 articles were identified, of which 20 articles met the inclusion criteria and were included in the qualitative analysis. The available evidence revealed that multi-joint strength values were limited to knee osteoarthritis, fibromyalgia, chronic low back pain, and rheumatoid arthritis. Only four studies were included in the quantitative synthesis and revealed that only small differences in both chest press (*g* = −0.34, 95% CI [−0.64, −0.03]) and leg press (*g* = −0.25, 95% CI [−0.49, −0.02]) existed between adult women with fibromyalgia and active community women.

**Conclusion:**

There is a paucity of multi-joint strength values in participants with musculoskeletal pain. Quantitative comparison with healthy cohorts was limited, except for those with fibromyalgia. Adult women with fibromyalgia displayed reduced multi-joint strength values in comparison to active community women.

## Introduction

The World Health Organization guidelines emphasize the importance of aerobic and resistance training to prevent non-communicable diseases in the general population.^1^ These are also recommended in clinical guidelines for spinal and peripheral musculoskeletal disorders.^2–6^ It is widely recognized that resistance training, and in particular strength training, provides multi-systemic and specific adaptations in healthy people and in a variety of musculoskeletal disorders.^7–10^ These include the risk reduction of cardiovascular diseases, type 2 diabetes, depression, dementia, colon cancer and postmenopausal breast cancer and also specific benefits on bone, cartilage, muscle and tendon properties.^7,10^ Evidence of the effectiveness of exercise, delivered within a biopsychosocial framework, in reducing persistent pain is robust.^8,11^ Recommendations from high-quality clinical practice guidelines for musculoskeletal pain include a careful strength assessment to ensure that rehabilitation programmes are patient-centred and tailored to the individual's needs.^12^

Dynamic strength assessment is commonly used to measure muscular performance throughout the entire range of motion.^13^ This has revealed ‘good’ to ‘excellent’ reliability using 1 repetition maximum testing, which is therefore considered the ‘gold standard’ test of dynamic strength.^14,15^ When using multiple repetition maximum (2–10 repetitions maximum) to estimate 1 repetition maximum, prediction accuracy remains high only within 5 repetition maximum.^13,16^ Multi-joint strength assessment is a reflection of global muscular strength.^13,17–19^ This commonly involves multiple joints and large muscle groups (e.g., back/front squat, leg press, bench press, chest press, deadlift, mid-thigh pull, overhead press).^17,18,20^ It is currently unknown whether global measures of strength are more strongly associated with pain and disability levels than single-joint strength measures,^21,22^ and primarily if people with musculoskeletal pain display different multi-joint strength capacities than healthy cohorts. Clinical trials demonstrated that people with musculoskeletal pain are capable to improve their strength when exposed to specific training interventions.^23,24^ Hence, baseline strength is an important variable to consider for clinicians aiming to achieve what is considered ‘strong enough’. This may influence the rehabilitation programme (e.g., duration, frequency, volume, intensity), and individual prescriptions are required to achieve the targeted strength gains.^18,25^ Furthermore, pain interference might reduce maximal voluntary contraction,^26–28^ and thus should be reported and taken into consideration when interpreting the results.^26,29,30^ Thus, the aim of this systematic review was to investigate whether people with musculoskeletal pain show differences in global measures of strength in comparison to healthy cohorts.

## Methods

### Protocol

The original protocol was registered in the International Prospective Register of Systematic Reviews (PROSPERO) on 21 April 2021 (registration number: CRD42021249647). The recommendations of the Preferred Reporting Items for Systematic Reviews and Meta-analyses (PRISMA) were followed to prepare, conduct, and report this review.^31^

### Eligibility criteria and information sources

The studies were selected according to the Participants, Intervention, Comparison, Outcome, and Study design framework (PICOS).^31^ Clinical trials investigating participants with musculoskeletal painful conditions and measuring strength via multi-joint exercises were considered. They had to be primary study design with original data, written in English language, and published in peer-reviewed journals. Studies involving participants with previous reconstructive surgical procedures were excluded. The outcome measures were multi-joint strength values measured at baseline, thus excluding confounding effects of any particular intervention. The additional inclusion criteria were (a) presence of predominant musculoskeletal painful disorder; (b) no previous reconstructive surgical procedure(s); (c) strength assessment via multi-joint exercise(s) measured at baseline.

Studies were excluded for the following reasons: (a) absence of predominant musculoskeletal painful disorder; (b) presence of non-musculoskeletal predominant disease (e.g., malignancy); (c) previous reconstructive surgical procedure(s); (d) absence of multi-joint strength values measured at baseline; (e) no conventional assessment of strength (e.g., manual muscle testing); (f) measures of muscular strength using > 5 repetition maximum.

### Searches and study selection

A comprehensive literature search of three electronic databases (MEDLINE, SPORTDiscus and CINHAL) was conducted on 6 August 2022. The reference lists of articles and grey literature were also scanned. A systematic research strategy was developed by the author (EV) following the PICOS framework,^31^ with the contribution of the other two authors (JH and LM). The search strategy used is listed in Appendix 1 (Supplemental file). The keywords associated with musculoskeletal painful conditions (e.g., ‘low back pain’, ‘neck pain, ‘knee pain’, ‘shoulder pain’, ‘tendinopathy etc) were combined with terms related to multi-joint exercises (e.g., ‘back squat’, ‘leg press’, ‘mid-thigh pull’, ‘deadlift’, ‘bench press’ etc), using the Boolean operator ‘AND’.

All titles and abstracts were independently screened by two authors (EV and LM) to identify relevant studies. Titles and abstracts investigating participants with musculoskeletal conditions, which included the assessment of strength via multi-joint exercises were considered. Full-text manuscripts of remaining eligible studies were evaluated for inclusion in this review. Eligibility criteria were used to screen the articles.

### Data extraction

Data were extracted from the included studies. The data extraction process was reviewed by two authors (EV and LM) and disagreements with regard to the selection criteria were discussed and resolved by consensus including three authors (EV, LM and JH). Demographic and anthropometric details including gender, population size, age, body mass, body mass index and type of condition were recorded from each included study. The variables that were extracted and recorded included the multi-joint exercise(s) and comparison used, type(s) of load, and unit(s) of measurement. For any data that were not obtainable from the original manuscript, an email request was sent to the corresponding author for the missing information. In case of no response within a couple of weeks, a second email was sent. In the case of missing answers, the study with the missing data was excluded.

### Assessment of level of evidence, quality, and risk of bias in individual studies and across studies

The level of evidence, methodological quality, and risk of bias of each included study were independently examined by two authors (EV and LM). The level of evidence and quality of research design for each included study were assessed through the Oxford Centre for Evidence-Based Medicine level of evidence tool.^32^ This has 5 levels of evidence, where level 1 indicates the highest category and level 5 is the lowest.^32^ Modified Downs and Black scale was used to examine the quality of studies. It is a reliable tool for appraising the quality not only of randomized controlled trials but also non-randomized studies.^33^ The highest total score is 16. Scores ≥ 12 are considered ‘high’ quality; scores of 10 or 11 ‘moderate’ quality; scores ≤ 9 ‘low’ quality.^34^ The risk of bias of the selected studies was assessed using the PEDro Scale, which is a valid and reliable rating tool.^35^ It consists of 11 items that consider sequence generation, allocation concealment, blinding processes, outcome data completeness, and selective outcome reporting.^35,36^ Scores of < 4 are considered ‘poor’, 4–5 are considered ‘fair’, 6–8 are considered ‘good’ and 9–10 are considered ‘excellent’.^35^

The presence of any conflict of interest, publication bias, time-lag bias, selective data reporting, location bias or funding resources was examined through a risk of bias assessment for each of the selected studies.

### Data synthesis

Due to the different data reporting of the outcomes measured in the included studies, effect sizes (Hedge's *g*) were calculated as the standardized mean difference with mean ± standard deviation and 95% confidence using Review Manager Software (RevMan 5.3; Cochrane Collaboration, Oxford, UK). Data were analysed using the strength values at baseline in participants with musculoskeletal painful condition(s) compared with the strength values of healthy cohorts if available. As an alternative, strength values from a large database of active community members were considered.^37^ These values were collected online from 2007 through a self-filling form, which included age range, gender, bodyweight, exercise, repetition maximum and load used. One of the authors (MC) had full access to the dataset. Data were filtered so that only active participants displaying similar age, bodyweight and testing method were matched with our clinical samples. Furthermore, the author (MC) assessed the normal distribution of the data (using the Kolmogorov–Smirnov test) and the presence of outliers. Cohen's scale was used to interpret pooled standardized mean difference, where 0.2 represents a small effect, 0.5 is a moderate effect, and 0.8 is a large effect. Heterogeneity between studies was evaluated through *I*^2^ statistics, the Cochrane Chi-square (χ^2^), and the between-study variance using the tau-square (τ^2^) at 95% CI. The categorization to rate the level of heterogeneity was the following: *I*^2^ = 0%, no heterogeneity; *I*^2^ = 1%–25%, low heterogeneity, not important; *I*^2^ = 26%–50%, moderate heterogeneity; *I*^2^ = 51%–75%, high heterogeneity, substantial; *I*^2^ = 76%–100%, considerable heterogeneity.^38^ If two or more studies examined the same musculoskeletal condition and assessed baseline strength using the same exercise and testing method, these were considered eligible for meta-analysis and were ordered in forest plots based on the effect size.^39^ If similar examinations adopted different measurement units (e.g., pounds), these were converted in kilograms. In addition, if a study included two or more homogeneous experimental groups, their baseline anthropometric characteristics and strength measures were combined.^40^ Our synthesis of the level of evidence of the studies included in the quantitative analysis was based on guidelines reported by van Tulder et al.,^41^ accounting for study quality and statistical homogeneity of the included studies in the data sets. The level of evidence was considered ‘strong’ if findings were consistent among multiple high-quality randomized controlled trials; ‘moderate’ if findings were consistent among multiple low-quality randomized controlled trials and/or controlled clinical trials, and/or one high-quality randomized controlled trial; ‘limited’ if findings were based on one low-quality randomized controlled trial and/or controlled clinical trial; ‘conflicting’ with the presence of inconsistent findings among multiple trials (randomized controlled trials and/or controlled clinical trials); ‘no evidence’ when there was no evidence from any randomized controlled trials or controlled clinical trials.^41^

## Results

### Study selection and search results

**Figure 1.**
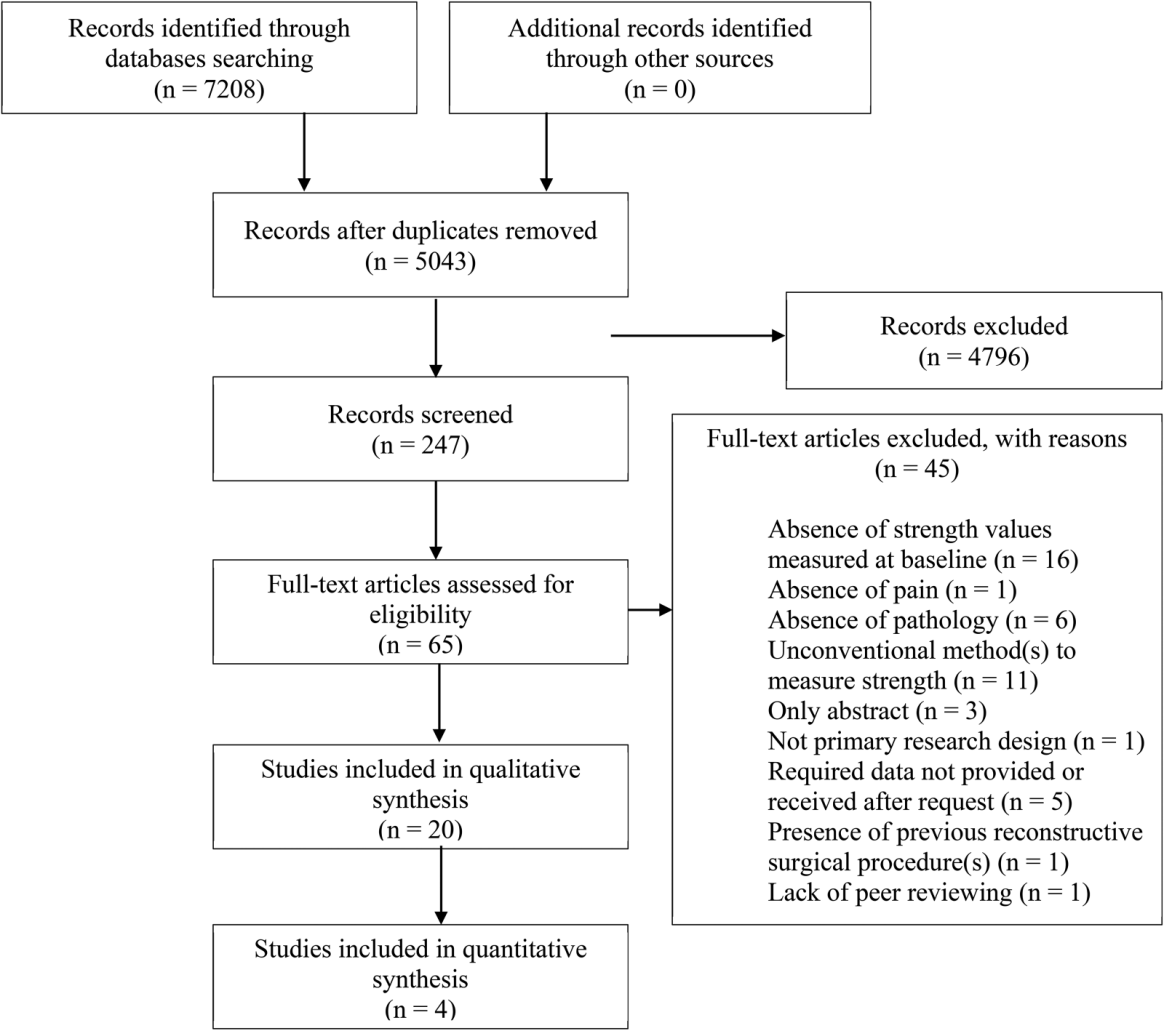
Preferred Reporting Items for Systematic Reviews and Meta-Analyses (PRISMA) flow diagram of included and excluded studies.

The initial electronic search identified 5043 articles from the databases (7208 before the duplicates were removed). After the evaluation of titles and abstracts, 4796 were excluded. Therefore, 247 articles were selected, and their titles and abstracts were further examined. 65 studies out of 247 were eligible and their full texts were obtained. Finally, 25 studies fulfilled the inclusion criteria and were included in the systematic review (Figure 1). Eight studies out of 25 had incomplete data (i.e., absence of clear and detailed strength values). Seven emails were sent to the corresponding authors to retrieve the missing data (one study did not display any email address). Only three authors out of seven answered the email request. Therefore, five articles out of 25 had to be excluded from the systematic review, leaving a final number of 20 included studies.

### Study characteristics

Participants and study characteristics are summarized in Table 1. Fifteen studies were randomized controlled trials, three were cross-sectional studies and two were single-arm trials. Ten studies analysed lower limb strength in participants with knee osteoarthritis using the bilateral leg press^42–49^ and unilateral leg press.^50,51^ Furthermore, four articles assessed upper limb strength in participants with fibromyalgia using the chest press^52–54^ and pectoral machine,^55^ while other two studies evaluated multi-joint strength in participants with fibromyalgia using both the chest press and the bilateral leg press.^56,57^ In addition, three studies assessed strength in participants with chronic low back pain with bilateral leg press, bench press and lat pulldown.^58–60^ Finally, one study analysed strength in women with rheumatoid arthritis using the leg press bilaterally.^61^

**Table 1.** Summary of the included studies.

Author, year and population studied	Participants (n), gender (W/M), age (yrs), BM and BMI	Interventions – strength assessment at baseline	Comparisons	Outcomes – Strength values measured at baseline	Study design
(1) Rodrigues et al., 2020	*n* = 48W	Leg press, 1RM, kg	No healthy controls	112.6 (33.0)	Randomized controlled trial
Adult women with rheumatoid arthritis (South America)	yrs = 58.6 (5.5)		Active community members		
	BM = 63.6 (12.3)				
	BMI = 26.3 (4.2)				
(2) Vincent et al., 2019	*n* = 90 (61W)	Leg press, 1RM, N・m	No healthy controls	602.7 (204.6)	Randomized controlled trial
Older adults with knee osteoarthritis (North America)	yrs = 68.2 (6.4)				
	BM = 86.2 (20.5)				
	BMI = 30.5 (6.8)				
			Active community members		
(3) Voigt et al., 2019	*n* = 7 (5M)	Leg press, 1RM, kg	No healthy controls	53.6 (8.6)	Single-arm trial
Older adults with knee osteoarthritis (North America)	yrs = 60–80		Active community members		
	BMI = < 30				
(4) Silva et al., 2019	*n* = 60W	Pectoral machine, 1RM, kg	No healthy controls	9.6 (5.3)	Randomized controlled trial
Mid-age women with fibromyalgia (South America)	yrs = 47.1 (9.5)		Active community members		
	BMI = 26.5 (6.9)				
(5) Pazit et al., 2018	*n* = 28 (15W)	Leg press, 3RM, kg/kg	No healthy controls	1.02 (0.59)	Randomized controlled trial
Older adults with knee osteoarthritis (Oceania)	yrs = 67.7 (6.4)		Active community members		
	BM = 84.4 (18.6)				
	BMI = 30.3 (6.8)				
(6) Ferraz et al., 2017	*n* = 48W	Leg press, 1RM, kg	No healthy controls	129.0 (42.5)	Randomized controlled trial
Older women with knee osteoarthritis (South America)	yrs = 60.3 (3.6)		Active community members		
	BM = 74.3 (12.6)				
	BMI = 30.1 (3.7)				
(7) Glasgow et al., 2017	*n* = 26W	Chest press, 1RM, kg	Healthy control group without strength assessment at baseline	28.5 (12.3)	Randomized controlled trial
Adult women with fibromyalgia (North America)	yrs = 52 (13)		Active community members		
	BM = 86 (17)				
	BMI = 32.5 (4.4)				
(8) Tevald et al., 2016	*n* = 40 (26W)	Leg press, 1RM, kg/kg	No healthy controls	1.7 (0.6)	Cross-sectional study
	yrs = 57 (10)				
	BMI = 35 (8)				
Adults with knee osteoarthritis (North America)			Active community members		
(9) Reid et al., 2015	*n* = 190 (132W)	Leg press, 1RM, N	No healthy controls	1338.5 (389.0) (M)	Cross-sectional study
Adults with knee osteoarthritis (North America)	yrs = 60.2 (10.4)		Active community members	835.6 (302.0) (W)	
	BM = 90.8 (21.0)				
	BMI = 32.7 (7.2)				
(10) Vincent et al., 2014	*n* = 49 (45W)	Leg press, 1RM, N・m	No healthy controls	330.2 (121.2)	Randomized controlled trial
Obese, older adults with chronic low back pain (North America)	yrs = 68.3 (6.8)		Active community members		
	BM = 91.6 (17.3)				
	BMI = 32.4 (4.8)				
(11) Gavi et al., 2014	*n* = 66W	Leg press, 1RM, kg	No healthy controls	107.5 (36.8)	Randomized controlled trial
Older women with fibromyalgia (South America)	yrs = 46.4 (8.0)		Active community members		
	BM = 66.7 (11.5)				
	BMI = 26.9 (4.5)				
(12) Sayers et al., 2012	*n* = 33 (17W)	Leg press, 1RM, N	No healthy controls	1371.6 (447.6)	Randomized controlled trial
Older adults with knee osteoarthritis (North America)	yrs = 67.1 (7.0)		Active community members		
	BM = 87.6 (20.1)				
	BMI = 30.6 (7.2)				
(13) Petersen et al., 2011	*n* = 36 (20W)	Leg press, 5RM, kg	No healthy controls	109.6 (14.0)	Randomized controlled trial
Older adults with knee osteoarthritis (Europe)	yrs = 62.3 (4.2)		Active community members		
	BM = 80.4 (15.2)				
	BMI = 27.7 (3.4)				
(14) Kell et al., 2011	n = 240 (156M)	Bench press – lat pulldown – leg press, 5RM, kg	No healthy controls	BP 53.8 (8.4)	Randomized controlled trial
Adults with chronic low back pain (North America)	yrs = 42.5 (6.0)		Active community members	LPu 54.5 (6.2)	
	BM = 78.2 (8.9)			LPr 136.1 (28.3)	
	BMI = 24.6 (5.0)				
(15) Jackson et al., 2011	*n* = 45M	Bench press – lat pulldown – leg press, 1RM (derived from 5RM), kg	No healthy controls	BP 70.7 (8.5)	Randomized controlled trial
Middle and older age men with chronic low back pain (North America)	yrs = 57.3 (6.7)		Active community members	LPu 56.3 (8.3)	
	BM = 78.2 (3.7)			LPr 126.7 (15.7)	
	BMI = 25.8 (3.6)				
(16) Panton et al., 2009	*n* = 21W	Chest press, 1RM, kg	No healthy controls	75.4 (17.7)	Randomized controlled trial
Adult women with fibromyalgia (North America)	yrs = 48.4 (9.8)		Active community members		
	BM = 81.7 (19.3)				
	BMI = 38.8 (7.0)				
(17) Kingsley et al., 2005	*n* = 29W	Chest press, 1RM, kg	No healthy controls	38.5 (11.9)	Randomized controlled trial
Adult women with fibromyalgia (North America)	yrs = 46.0 (6.8)		Active community members		
	BM = 84.3 (21.8)				
	BMI = 43.0 (7.4)				
(18) Rooks et al., 2002	*n* = 15W	Chest press – leg press, 1RM, pounds	No healthy controls	CP 61 (18) [27,6 (8,1) kg]	Single arm trial
Adult women with fibromyalgia (North America)	yrs = 44.9 (8.8)		Active community members	LPr 191(75) [86,6 (34) kg]	
(19) McNair et al., 2011	*n* = 18 (11W)	Unilateral leg press, 1RM, kg	No healthy controls	Involved limb 58.7 (25.9)	Cross-sectional study
Adults with knee osteoarthritis (Oceania)	yrs = 57 (10)		Active community members	Control limb 65.3 (30.2)	
	BM = 78 (13)				
	BMI = 27 (4)				
(20) Bily et al., 2019	*n* = 75 (51W)	Unilateral leg press (affected side), MVC - Isometric 90° knee flexion, N・m	No healthy controls	7.15 (3.07)	Cross-sectional study
Older adults with severe knee osteoarthritis (Europe)	yrs = 67.3 (7.3)		Active community members		
	BM = 81.2 (14.1)				
	BMI = 28.8 (4.4)				

*n*: number of participants; W: women; M: men; yrs: years; BM: body mass; BMI: body mass index; RM: repetition maximum; N・m: newton-metre; N: newton; kg: kilograms; kg/kg: kilograms/kilograms; MVC: maximum voluntary contraction; BP: bench press; LPu: lat pulldown; LPr: leg press; CP: chest press.

### Level of evidence, study quality and risk of bias

The Oxford Centre for Evidence-Based Medicine level of evidence tool, modified Downs and Black scores and PEDro scale for each study can be found in Tables 2 and 3. The Oxford Centre for Evidence-Based Medicine level of 15 studies was 2 (randomized controlled trials) and five were classified as 3 (single-arm trials and cross-sectional studies). The Downs and Black scores of the included studies ranged from 9 to 15. One study was of ‘low’ quality (i.e., 9), seven studies scored ‘moderate’ (i.e., 10–11), while the remaining 12 studies were considered of ‘high’ quality (i.e., 12–13–14–15). Ratings were examined by two authors (EV and LM) without any disagreement. PEDro scores varied across all 20 studies. Five studies had scores considered ‘poor’ (i.e., 3), three studies scored ‘fair’ (i.e., 4–5), 11 studies scored ‘good’ (i.e., 6–7), and only one study had scores considered ‘excellent’ (i.e., 9). All the authors agreed with the ratings.

**Table 2.** OCEBM level and modified Downs and Black scores of each study.

Modified Downs and Black score	1	2	3	4	5	6	7	8	9	10	11	12	13	14	15	Total Score	OCEBM level (lv)
Voigt (2019)	1	1	1	0	0	1	1	0	1	1	1	1	0	0	0	9	Lv 3
Reid (2015)	1	1	1	1	0	1	1	0	1	1	1	1	0	0	0	10	Lv 3
Panton (2009)	1	1	1	1	1	1	1	0	1	1	1	1	0	0	0	10	Lv 2
Kingsley (2005)	1	1	1	1	1	1	1	0	1	1	1	1	0	0	0	10	Lv 2
Rooks (2002)	1	1	1	1	1	1	1	0	1	1	1	1	0	0	0	10	Lv 3
Tevald (2016)	1	1	1	1	0	1	1	0	1	1	1	1	0	1	0	11	Lv 3
McNair (2011)	1	1	1	1	1	1	1	0	1	1	1	1	0	0	0	11	Lv 3
Bily (2019)	1	1	1	1	1	1	1	0	1	1	1	1	0	0	0	11	Lv 3
Pazit (2018)	1	1	1	1	1	1	1	0	1	1	1	1	0	1	0	12	Lv 2
Glasgow (2017)	1	1	1	1	1	1	1	0	1	1	1	1	0	1	1	13	Lv 2
Sayers (2012)	1	1	1	1	1	1	1	1	1	1	1	2	0	0	0	13	Lv 2
Kell (2011)	1	1	1	1	1	1	1	0	1	1	1	1	0	1	1	13	Lv 2
Jackson (2011)	1	1	1	1	1	1	1	0	1	1	1	1	0	1	1	13	Lv 2
Silva (2019)	1	1	1	1	1	1	1	1	1	1	1	1	0	1	1	14	Lv 2
Vincent (2014)	1	1	1	1	1	1	1	0	1	1	1	2	0	1	1	14	Lv 2
Gavi (2014)	1	1	1	1	1	1	1	1	1	1	1	2	0	0	1	14	Lv 2
Petersen (2011)	1	1	1	1	1	1	1	1	1	1	1	2	0	1	0	14	Lv 2
Rodrigues (2020)	1	1	1	1	1	1	1	1	1	1	1	2	0	1	1	15	Lv 2
Vincent (2019)	1	1	1	1	1	1	1	1	1	1	1	2	0	1	1	15	Lv 2
Ferraz (2017)	1	1	1	1	1	1	1	1	1	1	1	2	0	1	1	15	Lv 2

**Table 3.** PEDro score of each study.

PEDro scale	1	2	3	4	5	6	7	8	9	10	11	Total score
Voigt (2019)	√	X	X	X	X	X	X	√	√	X	√	3
Tevald (2016)	√	X	X	X	X	X	X	√	√	X	√	3
Reid (2015)	√	X	X	X	X	X	X	√	√	X	√	3
McNair (2011)	√	X	X	X	X	X	X	√	√	X	√	3
Bily (2019)	√	X	X	X	X	X	X	√	√	X	√	3
Kingsley (2005)	√	X	X	√	X	X	X	X	√	√	√	4
Rooks (2002)	√	X	X	√	X	X	X	X	√	√	√	4
Panton (2009)	√	√	X	√	X	X	X	X	√	√	√	5
Pazit (2018)	√	√	X	√	X	X	X	√	√	√	√	6
Ferraz (2017)	√	√	X	√	X	X	X	√	√	√	√	6
Glasgow (2017)	√	√	X	√	X	X	X	√	√	√	√	6
Sayers (2012)	√	√	X	√	X	X	√	X	√	√	√	6
Kell (2011)	√	√	X	√	X	X	X	√	√	√	√	6
Jackson (2011)	√	√	X	√	X	X	X	√	√	√	√	6
Rodrigues (2020)	√	√	X	√	X	X	√	√	√	√	√	7
Vincent (2019)	√	√	X	√	X	√	√	X	√	√	√	7
Silva (2019)	√	√	X	√	X	X	√	√	√	√	√	7
Vincent (2014)	√	√	X	√	X	√	X	√	√	√	√	7
Gavi (2014)	√	√	X	√	X	X	√	√	√	√	√	7
Petersen (2011)	√	√	X	√	√	√	√	√	√	√	√	9

The level of evidence of the included studies varied from 2 to 3 (Oxford Centre for Evidence-Based Medicine level). Moderate level of evidence indicating lower global level of strength was present in studies involving adult women with fibromyalgia, whereas conflicting evidence was available in studies investigating knee osteoarthritis, chronic low back pain and rheumatoid arthritis. Risk of bias assessment (based on PEDro scale) showed that the majority of the included studied scored ‘good’ (11), while others scored ‘poor’ (5) or fair (3) and only one was ‘excellent’. The most frequent sources of methodological considerations were: blinding of those measuring the main outcomes and administering or receiving the treatment(s), distribution and adjustment for confounders (e.g., gender), and sample size calculation. The distribution of the principal confounders was often described in the included studies (i.e., anthropometrics, physical activity levels, etc.). Most of the included studies showed clear eligibility criteria, adequate statistical analysis between groups for at least one key outcome and complete outcome measures.

Of the 20 studies included, 16 received funds or grants to support their research. No conflict of interest was reported in 12 studies, while the remaining studies omitted to clearly state this. There was no selective data reporting in all studies examined. Two articles were published ‘open access’.

### Findings of studies

Ten included studies involved adults with knee osteoarthritis (total *n* = 565). Of these, McNair et al.^51^ assessed 18 adults (11 women and 7 men) using the leg press unilaterally (1 repetition maximum and kilograms). Strength measures ranged from 58.7 (25.9) to 65.3 (30.2) kg for the affected and unaffected limb respectively. Voigt et al.^42^ and Ferraz et al.^43^ analysed 7 older adults (5 men and 2 women) and 48 older women respectively, using the bilateral leg press (1 repetition maximum and kilograms), and found values of 53.6 (8.6) and 129.0 (42.5) kg. In two different studies^44,45^ assessed bilateral leg press values (1 repetition maximum and newton) of 190 (132 women and 58 men) and 33 (17 women and 16 men) older adults respectively. Reid et al.^44^ displayed scores of 1338.5 (389.0) newton for men and 835.6 (302.0) newton for women, while Sayers et al.^45^ 1371.6 (447.6) newton. Vincent et al.^46^ measured lower limb strength in 90 older adults (61 women and 29 men) using the bilateral leg press (1 repetition maximum and newton-metre), and found torque values of 602.7 (204.6) newton-metre. Similarly, Tevald et al.^47^ used the bilateral leg press (1 repetition maximum and relative values) to assess strength in 40 adults (26 women and 14 men), showing a mean value of 1.7 (0.6) kg/kg. Pazit et al.^48^ measured lower limb strength in 28 older adults (15 women and 13 men) using the bilateral leg press (3 repetition maximum and relative values), showing values of 1.02 (0.59) kg/kg. Also, Petersen et al.^49^ used the bilateral leg press (5 repetition maximum and kilograms) to assess 36 older adults (20 women and 16 men) and found strength values of 109.6 (14.0) kg. Finally, Bily et al.^50^ analysed isometric lower limb strength (at 90° knee flexion) of 75 older adults (51 women and 24 men) using the leg press unilaterally (maximal voluntary contraction and newton-metre, affected side), and found a mean value of 7.15 (3.07) newton-metre.

Six included studies assessed adult women presenting with fibromyalgia (total *n* = 217). Of these, Glasgow et al.,^52^ and Kingsley et al.^54^ assessed 26 and 15 adult women (using the chest press, 1 repetition maximum and kilograms) respectively. Strength values were 28.5 (12.3) and 38.5 (11.9) kg, respectively. Also Panton et al.^53^ measured upper limb strength in 21 adult women, albeit with an unconventional weight machine (MedX™ Chest Press, thus excluded from the quantitative analysis), and found a mean value of 75.4 (17.7) kg. De Almeida Silva et al.^55^ assessed 60 middle-aged women using the pectoral machine (1 repetition maximum and kilograms) and found a mean value of 9.6 (5.3). In addition, Gavi et al.^56^ analysed lower limb strength in 66 older adult women using the leg press bilaterally (1 repetition maximum and kilograms) and showing a value equal to 107.5 (36.8) kg. Lastly, Rooks et al.^57^ measured strength in 15 adult women using the chest press and leg press (1 repetition maximum and pounds), and found values of 61 (18) pounds (i.e., 27,6 (8,1) kg) and 191 (75) pounds (i.e., 86,6 (34) kg), respectively.

Three included studies analysed adults with chronic low back pain (total *n* = 334). Jackson et al.^60^ assessed 45 middle-aged and older men using the bench press, lat pulldown and bilateral leg press (1 repetition maximum derived from 5 repetition maximum and kilograms). Bench press strength values were 70.7 (8.5) kg, lat pulldown were 56.3 (8.3) kg, and leg press were 126.7 (15.7) kg. Differently, Kell et al.^59^ measured multi-joint strength in 240 adults (151 men and 89 women) using the bench press, lat pulldown and bilateral leg press (5 repetition maximum and kilograms). Strength scores of bench press were 53.8 (8.4) kg, lat pulldown were 54.5 (6.2) kg, and leg press were 136.1 (28.3) kg. Finally, Vincent et al.^58^ evaluated 49 obese older adults (45 women and 4 men) using the bilateral leg press (1 repetition maximum and newton-metre) and showing strength values of 330.2 (121.2) newton-metre.

Only one study^61^ assessing strength in adult women with rheumatoid arthritis (total *n* = 48) met our inclusion criteria. This used the bilateral leg press (1 repetition maximum) and found a mean baseline value of 112.6 (33.0) kg.

### Synthesis of findings

**Figure 2.**
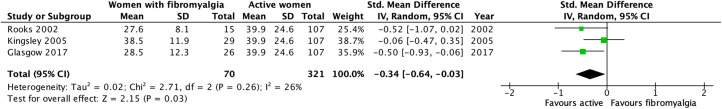
Forest plot for strength values using chest press, 1 repetition maximum and kilograms comparing women with fibromyalgia with active community women.

**Figure 3.**
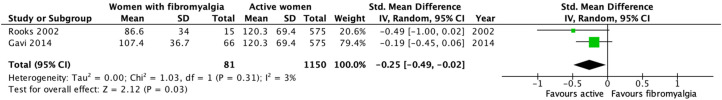
Forest plot for strength values using leg press, 1 repetition maximum and kilograms comparing women with fibromyalgia with active community women.

Due to the different conditions, exercises, loads and units of measurements used to assess strength at baseline, only four of the 20 studies were deemed eligible for inclusion in the meta-analysis (total participants *n* = 151).^52,54,56,57^ These studies measured baseline strength values in women with fibromyalgia. Three of them used chest press (1 repetition maximum),^52,54,57^ while two studies assessed strength with the bilateral leg press (1 repetition maximum).^56,57^ None of these studies had a healthy control group. Therefore, the authors used values from a large dataset of active community members,^37^ matching the age mean and standard deviation, gender(s), exercise(s) and testing method (i.e., 1 repetition maximum) specified in the studies. The pooled results of strength values using these multi-joint exercises are presented in Figures 2 and 3.

### Chest press (1 repetition maximum) in women with fibromyalgia

Pooled data showed moderate evidence indicating a small negative effect (*g* = −0.34, 95% CI [−0.64, −0.03]; *I*^2^ = 26%) of fibromyalgia on upper limb strength in women with fibromyalgia compared to active community women.

### Bilateral leg press (1 repetition maximum) in women with fibromyalgia

Pooled data showed moderate evidence indicating a small negative effect (*g* = −0.25, 95% CI [−0.49, −0.02]; *I*^2^ = 3%) of fibromyalgia on lower limb strength in women with fibromyalgia compared to active community women.

## Discussion

The aim of this review was to synthesize and critically evaluate the available literature assessing multi-joint strength values in participants with musculoskeletal painful conditions, and to investigate whether these differ from healthy cohorts. The main findings revealed that, although clinical guidelines recommend strength assessment as an essential part of physical examination in participants presenting with musculoskeletal pain disorders,^12^ only very limited data is available on multi-joint strength levels. These were limited to knee osteoarthritis, fibromyalgia, chronic low back pain and rheumatoid arthritis. However, owing to the heterogeneous samples, varied and inaccurate strength assessment methods among the included trials, and absence of healthy active control groups, it was not possible to determine whether participants with knee osteoarthritis, chronic low back pain and rheumatoid arthritis presented with different multi-joint strength levels than healthy active cohorts. A meta-analytic review was available only for women with fibromyalgia since it is recommended to include at least two adequately homogeneous studies for quantitative analysis.^62^ However, controls were extracted from a database of active community members, with limitations highlighted in the specific section of this review. Interestingly, no measures of multi-joint strength level were reported in sports rehabilitation research (e.g., upper and lower limb tendinopathies cohorts), where thorough physical preparedness is often advocated before return to sport.^63–65^

Strength was measured with free weights only in two studies with the same exercise (i.e., bench press).^59,60^ All other included studies assessed strength with weight machines (i.e., leg press, chest press, lat pulldown and pectoral machine). While the choice of weights machines was fairly consistent in our review, the included studies presented heterogeneity in strength measurement testing methods. The American College of Sports Medicine guidelines for exercise testing and prescription^13^ suggests that dynamic strength should be measured with 1 repetition maximum^13–15^ or multiple repetition maximum ( ≤ 5) to provide a fair reflection of individual strength.^13^ However, poor knowledge of exercise physiology principles in musculoskeletal research and healthcare professionals may be a barrier to implementing such recommendations, and further professional training may be needed.^66–68^ Strength levels vary significantly between men and women,^69–71^ and thus cohorts should be divided into separate groups according to their gender. In our review, 10 studies included mixed groups not accounting for gender differences.^42,45–51,58,59^ Poor reporting of relevant participants characteristics clearly limits the external validity of the findings (i.e., their generalizability or applicability into clinical practice), and accurate systematic synthesis of the available literature. This has been also identified in tendinopathy and chronic low back pain research,^72–74^ with better depiction of participants characteristics in clinical studies being recommended in the CONSORT statement.^75^

The influence of experimental and clinical pain on muscle activity and maximal voluntary contraction has been widely investigated.^26–30^ The available evidence suggests that pain reduces strength capabilities in participants with persistent pain conditions.^26,28,76,77^ Only one study included in this systematic review reported whether participants (namely 6 participants out of 18) could not perform the 1 repetition maximum strength test due to pain severity.^51^ None of the remaining studies mentioned pain interference during multi-joint strength testing. Therefore, it may be inferred that pain interference was not a barrier during multi-joint strength assessment in the included samples. However, more clarity is needed when describing physical performance testing in participants with musculoskeletal pain disorders.

Only four studies assessing adult women with fibromyalgia were deemed eligible for inclusion in our meta-analysis.^52,54,56,57^ Our results (moderate level of evidence) suggested that only small differences in strength were present between women with fibromyalgia and active peers in both multi-joint exercises examined (i.e., chest press and leg press). However, caution should be taken when interpreting these results. Three out of four studies^54,56,57^ used outdated criteria to identify participants with fibromyalgia^78^ (e.g., tender points examination), which may overestimate the presence of such a condition.^79–81^ This may result in the erroneous inclusion of participants presenting with nociceptive musculoskeletal pain with signs typical of central pain processes (i.e., increased pain response evoked by stimuli outside the affected area).^82–84^ Therefore, further studies identifying participants with fibromyalgia using the most appropriate diagnostic criteria and assessing strength with multi-joint exercises are needed to corroborate our results.

Baseline bilateral leg press strength values are available in healthy adult women cohorts.^85,86^ These did not show significant differences in comparison with adult women with rheumatoid arthritis and knee osteoarthritis included in our review.^43,61^ Conversely, leg press strength values of healthy adult men^87^ appeared to show significant differences with adult men with persistent low back pain.^60^ However, owing to the heterogeneous participants' characteristics, further studies with healthy matched controls are needed to draw clear conclusions.

None of the included studies compared multi-joint strength levels of participants with musculoskeletal pain with healthy controls at baseline. Therefore, we used a large database of strength values obtained from active community members to provide a suitable comparison group for the quantitative analysis where indicated. Although the large sample is one strength of the database used, past and current medical records of each individual are not available, which cannot confirm the pain-free status of our active controls. Research shows that people with persistent pain have more difficulties in engaging in positive lifestyle behaviours and are more physically inactive than healthy cohorts.^88,89^ Therefore, it may be speculated that our active cohorts may be a valid comparison, although a more robust methodology is needed. Furthermore, although strength values were entered in the database by the active individuals directly, the values obtained from our active cohort were not different from published research where investigators collected the data.^85,86,90,91^ This may indicate that testing using simple and commercially available weight machines is fairly simple, albeit careful quality checking to ensure guidelines are followed^13,92^ during strength assessment is recommended in further research.

The findings from this systematic review and meta-analysis revealed a paucity of multi-joint strength data in people with painful musculoskeletal conditions. The available evidence was limited to knee osteoarthritis, fibromyalgia, chronic low back pain and rheumatoid arthritis. Generalizability was also limited by the heterogeneous samples and testing methods included. These discrepancies, together with the absence of healthy active controls did allow for quantitative comparison with healthy cohorts, only for fibromyalgia. Adult women with fibromyalgia displayed reduced multi-joint strength values in comparison to active women. More robust research is needed to clarify whether people with musculoskeletal pain display different multi-joint strength levels than healthy cohorts.

**Clinical messages**

- Collect strength measurements as recommended by clinical guidelines to increase their accuracy.
- Account for gender and age differences when conducting strength assessments.
- Comparing strength measurements with valid reference values can help to improve individual rehabilitation programme prescriptions.
- Report if pain occurs during testing procedures since this can reduce strength.

## Supplemental Material

sj-docx-1-cre-10.1177_02692155221128724 - Supplemental material for Do people with musculoskeletal pain differ from healthy cohorts in terms of global measures of strength? A systematic review and meta-analysis Click here for additional data file. Supplemental material, sj-docx-1-cre-10.1177_02692155221128724 for Do people with musculoskeletal pain differ from healthy cohorts in terms of global measures of strength? A systematic review and meta-analysis by Enrico Verdini, Luca Maestroni, Michael Clark, Anthony Turner and Jörg Huber in Clinical Rehabilitation
