# Supplementary material for: Do people with musculoskeletal pain differ from healthy cohorts in terms of global measures of strength? A systematic review and meta-analysis
Source: Clin Rehabil. 2022 Sep 25;37(2):244–60. doi: 10.1177/02692155221128724 (PMC9772898; doi:10.1177/02692155221128724)
Supplement: sj-docx-1-cre-10.1177_02692155221128724 - Supplemental material for Do people with musculoskeletal pain differ from healthy cohorts in terms of global measures of strength? A systematic review and meta-analysis [file sj-docx-1-cre-10.1177_02692155221128724.docx]

**APPENDIX 1**

**SEARCH TERMS, STRATEGIES, AND RESULTS**

**Date: Saturday, August 6, 2022**

| **Search ID#** | **Search Terms** | **Search options** | **Last run via** | **Results** |
| --- | --- | --- | --- | --- |
| **S1** | pain OR nonmalignant pain OR persistent pain OR chronic pain OR back pain OR low backpain OR neck pain OR knee pain OR shoulder pain OR hip pain OR elbow pain OR shoulder pain OR ankle pain OR foot pain OR wrist pain OR thoracic pain OR hand pain OR anterior cruciate ligament tear OR ligament tear* OR muscle tear* OR anterior cruciate ligament reconstruction OR pubalgia OR groin pain OR groin strain* OR adductor syndrome OR insertional tend* OR discogenic pain OR discogenic back pain OR facet* joint pain OR facet* joint syndrome OR cervicogenic headache OR sciatica OR arthritis OR osteoarthritis OR musculoskeletal pain OR muscle pain OR myalgia OR fibromyalgia OR tend* OR heel pain OR bursitis OR neuropathic pain OR musculoskeletal painful condition* OR musculoskeletal painful disorder* OR musculoskeletal painful disease* OR iliotibial band syndrome OR patellofemoral pain syndrome OR tibiofemoral pain syndrome OR chondr* OR nociceptive pain OR femoroacetabular impingement OR shoulder impingement syndrome OR subacromial impingement syndrome OR lateral epicondylitis OR myofascial pain syndrome OR plantar fasci* | **Limiters** – Peer Reviewed; English Language; Human; English Language; Human; Language: English  **Expanders** – Apply equivalent subjects  **Search modes** - Boolean/Phrase | **Interface -** EBSCOhost Research Databases  **Search Screen –** Advanced Search  **Database -** MEDLINE;CINAHL;SPORTDiscus | 1,355,717 |
| **S2** | strength training OR resistance training OR squat OR back squat OR front squat OR leg press OR leg curl OR push up OR deadlift OR mid-thigh pull OR bench press OR overhead press OR overhead squat OR military press OR shoulder press OR lat pulldown OR pull up OR multi-joint exercise* | **Limiters** – Peer Reviewed; English Language; Human; English Language; Human; Language: English  **Expanders** – Apply equivalent subjects  **Search modes** - Boolean/Phrase | **Interface -** EBSCOhost Research Databases  **Search Screen –** Advanced Search  **Database -** MEDLINE;CINAHL;SPORTDiscus | 52,688 |
| **S3** | S1 AND S2 NOT SYSTEMATIC REVIEW | **Limiters -** PeerReviewed  **Expanders –** Apply equivalent subjects  **Search modes -** Boolean/Phrase | **Interface -** EBSCOhost Research Databases  **Search Screen –** Advanced Search  **Database -** MEDLINE;CINAHL;SPORTDiscus | 7,208 |

**PEDro scale**

| **PEDro scale** | 1 | 2 | 3 | 4 | 5 | 6 | 7 | 8 | 9 | 10 | 11 | Total Score |
| --- | --- | --- | --- | --- | --- | --- | --- | --- | --- | --- | --- | --- |
| Voigt (2019) | √ | X | X | X | X | X | X | √ | √ | X | √ | 3 |
| Tevald (2016) | √ | X | X | X | X | X | X | √ | √ | X | √ | 3 |
| Reid (2015) | √ | X | X | X | X | X | X | √ | √ | X | √ | 3 |
| McNair (2011) | √ | X | X | X | X | X | X | √ | √ | X | √ | 3 |
| Bily (2019) | √ | X | X | X | X | X | X | √ | √ | X | √ | 3 |
| Kingsley (2005) | √ | X | X | √ | X | X | X | X | √ | √ | √ | 4 |
| Rooks (2002) | √ | X | X | √ | X | X | X | X | √ | √ | √ | 4 |
| Panton (2009) | √ | √ | X | √ | X | X | X | X | √ | √ | √ | 5 |
| Pazit (2018) | √ | √ | X | √ | X | X | X | √ | √ | √ | √ | 6 |
| Ferraz (2017) | √ | √ | X | √ | X | X | X | √ | √ | √ | √ | 6 |
| Glasgow (2017) | √ | √ | X | √ | X | X | X | √ | √ | √ | √ | 6 |
| Sayers (2012) | √ | √ | X | √ | X | X | √ | X | √ | √ | √ | 6 |
| Kell (2011) | √ | √ | X | √ | X | X | X | √ | √ | √ | √ | 6 |
| Jackson (2011) | √ | √ | X | √ | X | X | X | √ | √ | √ | √ | 6 |
| Rodriguez (2020) | √ | √ | X | √ | X | X | √ | √ | √ | √ | √ | 7 |
| Vincent (2019) | √ | √ | X | √ | X | √ | √ | X | √ | √ | √ | 7 |
| Silva (2019) | √ | √ | X | √ | X | X | √ | √ | √ | √ | √ | 7 |
| Vincent (2014) | √ | √ | X | √ | X | √ | X | √ | √ | √ | √ | 7 |
| Gavi (2014) | √ | √ | X | √ | X | X | √ | √ | √ | √ | √ | 7 |
| Petersen (2011) | √ | √ | X | √ | √ | √ | √ | √ | √ | √ | √ | 9 |

PEDro scores of the included studies (<https://www.pedro.org.au/english/downloads/pedro-scale/>)

**Modified Downs and Black scores**

| **Modified Downs and Black score** | 1 | 2 | 3 | 4 | 5 | 6 | 7 | 8 | 9 | 10 | 11 | 12 | 13 | 14 | 15 | Total Score | **OCEBM level (lv)** |
| --- | --- | --- | --- | --- | --- | --- | --- | --- | --- | --- | --- | --- | --- | --- | --- | --- | --- |
| Voigt (2019) | 1 | 1 | 1 | 0 | 0 | 1 | 1 | 0 | 1 | 1 | 1 | 1 | 0 | 0 | 0 | 9 | Lv 3 |
| Reid (2015) | 1 | 1 | 1 | 1 | 0 | 1 | 1 | 0 | 1 | 1 | 1 | 1 | 0 | 0 | 0 | 10 | Lv 3 |
| Panton (2009) | 1 | 1 | 1 | 1 | 1 | 1 | 1 | 0 | 1 | 1 | 1 | 1 | 0 | 0 | 0 | 10 | Lv 2 |
| Kingsley (2005) | 1 | 1 | 1 | 1 | 1 | 1 | 1 | 0 | 1 | 1 | 1 | 1 | 0 | 0 | 0 | 10 | Lv 2 |
| Rooks (2002) | 1 | 1 | 1 | 1 | 1 | 1 | 1 | 0 | 1 | 1 | 1 | 1 | 0 | 0 | 0 | 10 | Lv 3 |
| Tevald (2016) | 1 | 1 | 1 | 1 | 0 | 1 | 1 | 0 | 1 | 1 | 1 | 1 | 0 | 1 | 0 | 11 | Lv 3 |
| McNair (2011) | 1 | 1 | 1 | 1 | 1 | 1 | 1 | 0 | 1 | 1 | 1 | 1 | 0 | 0 | 0 | 11 | Lv 3 |
| Bily (2019) | 1 | 1 | 1 | 1 | 1 | 1 | 1 | 0 | 1 | 1 | 1 | 1 | 0 | 0 | 0 | 11 | Lv 3 |
| Pazit (2018) | 1 | 1 | 1 | 1 | 1 | 1 | 1 | 0 | 1 | 1 | 1 | 1 | 0 | 1 | 0 | 12 | Lv 2 |
| Glasgow (2017) | 1 | 1 | 1 | 1 | 1 | 1 | 1 | 0 | 1 | 1 | 1 | 1 | 0 | 1 | 1 | 13 | Lv 2 |
| Sayers (2012) | 1 | 1 | 1 | 1 | 1 | 1 | 1 | 1 | 1 | 1 | 1 | 2 | 0 | 0 | 0 | 13 | Lv 2 |
| Kell (2011) | 1 | 1 | 1 | 1 | 1 | 1 | 1 | 0 | 1 | 1 | 1 | 1 | 0 | 1 | 1 | 13 | Lv 2 |
| Jackson (2011) | 1 | 1 | 1 | 1 | 1 | 1 | 1 | 0 | 1 | 1 | 1 | 1 | 0 | 1 | 1 | 13 | Lv 2 |
| Silva (2019) | 1 | 1 | 1 | 1 | 1 | 1 | 1 | 1 | 1 | 1 | 1 | 1 | 0 | 1 | 1 | 14 | Lv 2 |
| Vincent (2014) | 1 | 1 | 1 | 1 | 1 | 1 | 1 | 0 | 1 | 1 | 1 | 2 | 0 | 1 | 1 | 14 | Lv 2 |
| Gavi (2014) | 1 | 1 | 1 | 1 | 1 | 1 | 1 | 1 | 1 | 1 | 1 | 2 | 0 | 0 | 1 | 14 | Lv 2 |
| Petersen (2011) | 1 | 1 | 1 | 1 | 1 | 1 | 1 | 1 | 1 | 1 | 1 | 2 | 0 | 1 | 0 | 14 | Lv 2 |
| Rodriguez (2020) | 1 | 1 | 1 | 1 | 1 | 1 | 1 | 1 | 1 | 1 | 1 | 2 | 0 | 1 | 1 | 15 | Lv 2 |
| Vincent (2019) | 1 | 1 | 1 | 1 | 1 | 1 | 1 | 1 | 1 | 1 | 1 | 2 | 0 | 1 | 1 | 15 | Lv 2 |
| Ferraz (2017) | 1 | 1 | 1 | 1 | 1 | 1 | 1 | 1 | 1 | 1 | 1 | 2 | 0 | 1 | 1 | 15 | Lv 2 |

Modified Downs and Black Score assesses study quality based on a set of 15 questions, with a total score of 16.

1 = Hypothesis/aim/objective of the study clearly described,

2 = Characteristics of the patients clearly described,

3 = Patient sample representative of patients treated in routine clinical practice,

4 = Is there information on possibility of selection bias,

5 = Was a comparison group identified, clearly defined,

6 = Are the main outcomes clearly described in the Introduction or Methods,

7 = Were the main outcome measures used accurate (valid and reliable),

8 = Any attempt to blind those measuring the main outcomes,

9 = Are the main findings of the study clearly described,

10 = Does the study provide estimates of the random variability,

11 = Were the statistical tests used to assess the main outcomes appropriate,

12 = Are the distribution of principal confounders in each group of participants to be compared clearly described,

13 = Was there adequate adjustment for confounding in the analyses from which the main findings were drawn,

14 = Was a sample size calculation reported,

15 = Sufficient power to detect a clinically important effect where the probability value for a difference being due to chance is less than 5%

†12 had score options of 2 = fully described and 1 = partially described

**Included dataset of active members of the community**

| **Gender** | **Exercise** | **Participants (n)** | **Mean absolute 1RM (kg)** | **Absolute 1RM (SD)** | **Relative 1 RM (kg kg^-1^)** | **Relative 1 RM (SD)** | **Mean age (years)** | **Age (SD)** |
| --- | --- | --- | --- | --- | --- | --- | --- | --- |
| Female | Leg press horizontal | 1148 | 120.35 | 69.43 | 1.71 | 0.90 | 49.19 | 6.58 |
| Female | Chest press | 320 | 39.98 | 24.61 | 0.58 | 0.35 | 50.97 | 7.53 |
